# Modulatory Effect of Competitive Exclusion on the Transmission of *ESBL E. coli* in Chickens

**DOI:** 10.1007/s12602-023-10095-1

**Published:** 2023-06-06

**Authors:** Amera F. Ebrahem, Azza S. El-Demerdash, Rania M. Orady, Nehal M. Nabil

**Affiliations:** 1 Agricultural Research Center, Reference Laboratory for Veterinary Quality Control On Poultry Production, Animal Health Research Institute, P.O. 12618, Gamasa, Egypt; 2 Agriculture Research Center (ARC), Animal Health Research Institute (AHRI), P.O. 44516, Zagazig, Egypt

**Keywords:** Competitive exclusion, Broiler chicken, Extended-spectrum β-lactamases, *Escherichia coli*, Anti-virulence property

## Abstract

The extensive use of antimicrobial agents in broiler farms causes the emergence of antimicrobial resistance of *E. coli* producing severe economic losses to the poultry industry; therefore, monitoring the transmission of ESBL *E. coli* is of great significance throughout broiler farms. For this reason, we investigated the efficiency of competitive exclusion (CE) products to control the excretion and transmission of ESBL-producing *E. coli* in broiler chickens. Three hundred samples from 100 broiler chickens were screened for the incidence of *E. coli* by standard microbiological techniques. The overall isolation percentage was 39% and differentiated serologically into ten different serotypes: O158, O128, O125, O124, O91, O78, O55, O44, O2, and O1. The isolates represented absolute resistance to ampicillin, cefotaxime, and cephalexin. The effectiveness of CE (commercial probiotic product; Gro2MAX) on ESBL-producing *E. coli *(O78) isolate transmission and excretion was studied in vivo. The results showed that the CE product has interesting properties, making it an excellent candidate for targeted drug delivery by inhibiting bacterial growth and downregulating biofilm, adhesins, and toxin-associated genes loci. The histopathological findings demonstrated the capability of CE in repairing internal organ tissues. Our outcomes suggested that the administration of CE (probiotic products) in broiler farms could be a safe and alternative approach to control the transmission of ESBL-producing virulent *E. coli* in broiler chickens.

## Introduction

Avian pathogenic *Escherichia coli* (APEC) causes colibacillosis that can be localized or systemic and is considered a potential foodborne zoonotic bacterium [1].

The infection starts with respiratory manifestations, septicemia, perihepatitis, and pericarditis in broiler chickens [2]. It is usually involved in high morbidity and mortality with significant economic losses for poultry farmers [3]. Numerous APEC serotypes are associated with these diseases, and each has some virulence characteristics that contribute to infection, such as toxin generation, biofilm formation, hemolysins, adhesion, and cell surface hydrophobicity [4].

In addition to the responsibility of the acquisition of virulence genes and their influence on pathogenicity, the acquirement of resistance genes plays a vital role in therapeutic disappointment and increases the mortality rate. According to the World Health Organization, one of the main threats to food security, development, and global health is antibiotic resistance [5]. Enzymatic inactivation of antibiotics by beta-lactamases is one of the bacterial resistance mechanisms. Extended-spectrum β-lactamase (ESBL) enzymes can hydrolyze β-lactam antibiotics, including third-generation cephalosporins [6].

The presence of ESBLs producing commensal enterobacteria in farm animals is one of the possible sources of food contamination [7]. In broiler and poultry production chains, there has been a significant incidence of ESBL producers and a high level of variation in ESBL genes [8]. The most prevalent ESBL-encoding genes among *E. coli* isolates from broiler farms are the *bla*_TEM_, *bla*_SHV_, and *bla*_CTX-M_ genes[9]

Many strategies have been proposed to reduce the prevalence of antimicrobial resistance, including non-antibiotic feed additives and CE products [10–12].

CE culture implementation at an early stage is essential to prevent beta-lactamase-producing *E. coli* colonization in newly hatched birds [8] and diminishing the level of ESBL-producing *E. coli* in broiler intestines [6]**.**

Therefore, we modeled the ingestion and colonization of chickens with ESBL-producing *E. coli* and validate the efficacy of commercial CE products to reduce this infection process experimentally and genotypically.

## Material and Methods

### Sample Collection, Preparation, and Isolate Identification

One hundred diseased broiler chickens (age varied from 30 to 37 days) were collected randomly from 20 different farms located in Dakahlia Governorate, Egypt. The collected birds suffered from diarrhea and lesions of coli-septicemia (colicystitis and air saculitis). Samples from internal organs such as the lungs, liver, and heart were collected aseptically and pooled as one sample from each bird individually. The samples were transported immediately to Reference Laboratory for Veterinary Quality control on Poultry production (RLQP) (Gamasa Lab.) for further examinations.

All of the collected samples were subjected to *E. coli* isolation and identification according to Quinn et al. [13]. The confirmed isolates were serotyped using known antisera (Sifin) concurring to Lee et al. [14]

### Antimicrobial Susceptibility Testing

The Kirby-Bauer disk diffusion assay was applied to determine the antimicrobial susceptibility pattern of isolates [15]. The confirmed *E. coli* isolates were tested against ten commonly used antimicrobial agents in Egyptian broiler farms. The involved antimicrobial disks were as follows amoxicillin (AM, 10 µg), ampicillin (AMP, 10 µg), cefotaxime (CTX, 30 µg), cephalexin (CL, 30 µg), streptomycin (S, 10 µg), tetracycline (TE, 30 µg), colistin sulfate (CT, 10 µg), neomycin (N, 30 µg), and sulfamethoxazole-trimethoprim (SXT, 23.75 μg). The methodology and data interpretations were carried out following CLSI [16]. *E. coli *ATCC 25922 was used as a control.

### Detection of bla_***TEM***_ Gene in the Isolated *E. coli* Using Conventional PCR Technique

The extraction of DNA was done following the manufacturer ‘s instructions QIAamp DNA Mini kits (Qiagen, Germany, GmbH, Catalogue no. 51304). The utilized primers included the following sequences: F; ATCAGCAATAAACCAGC and R; CCCCGAAGAACGTTTTC. The carried-out PCR cycling was in a final volume of 25 µl containing 12.5 µl of DreamTaq Green PCR Master Mix (2X) (Thermo Scientific), 1 µl of each primer of 20 pmol concentration, 5.5 µl of water, and 5 µl of DNA template. The performed reaction was in an Applied biosystem 2720 thermal cycler. The cycling conditions were 94 °C for 5 min followed by 35 cycles of initial denaturation at 94 °C for 30 s, annealing at 54 °C for 40 s, and extension of 72 °C for 7 min [17]. The previously confirmed positive field strain from the Reference Laboratory for Veterinary Quality Control on Poultry Production, Animal Health Research Institute, was used as a positive control and a negative control (PCR mixture without DNA template) to establish the PCR test's accuracy.

### Identification and Recognition of Challenged *E. coli* Isolate Based on Sequencing Assay

By sequencing the 16S rRNA gene, the challenged isolate was recognized. With prior procedures, universal primers 27F and 1492R were employed to amplify virtually full-length 16S rRNA gene sequences [18]. To segregate the DNA after amplification, it was electrophoresed on an agarose gel and stained with ethidium bromide (0.5 g/mL). When the PCR product was produced correctly, the precise 16S rRNA sequence could be utilized with forward and reverse primers. Sequencing of the PCR amplicons was done on an Applied Biosystems 3130 automated DNA Sequencer (ABI, 3130, USA) using one cycle sequencing kit (Perkin-Elmer/Applied Biosystems, Foster City, CA) with Cat. No. 4336817 and a ready reaction Bigdye Terminator V3.1 cycle sequencing kit of Cat. No. 4337455. BLAST software was employed to establish sequence identity to GenBank accession.

### In Vivo Assay of Competitive Exclusion Effect to Reduce ESBL-Producing *E. coli* Excretion and Transmission in Experimentally Infected Chicks

#### Birds

About 50 Ross broiler chicks (1 day old) obtained from a commercial hatchery in Dakahlia Governorate, Egypt, were placed in separate cages (biosecurity level, two).

#### Competitive Exclusion Culture Product (CE)

A commercial product of CE (Gro2MAX, LOT NO. 3202018G2M, BioNatural America, USA) (probiotic health supplement for poultry) was utilized for the experimental chicks. The composition of the product was rice bran, soybean meal, dried *Bacillus* *subtilis* fermentation product (7 × 10^6^ CFU/gm), dried *Lactobacillus* *acidophilus* fermentation product (3 × 10^6^ CFU/gm), dried *Pediococcus acidilactici* fermentation product (2 × 10^4^ CFU/gm), dried *Pediococcus pentosaceus* fermentation product (2 × 10^4^ CFU/gm), dried *Saccharomyces cerevisiae* fermentation product (1 × 10^6^ CFU/gm), and salt. Gro2MAX product was prepared as manufacture instructions as follows: 3.5 gm was mixed with a gallon of drinking water, and then 0.5 ml was applied orally using a needle-less sterile syringe on the 1st day of age in experimental groups (1, 2, and 3). The Gro2MAX product was tested for the absence of ESBL-producing *E. coli* as follows: 1 gm was suspended in 9 ml buffered peptone water plated on MacConkey agar plates containing cefotaxime (1 mg/liter agar).

#### Experimental Design

**Fig. 1:**
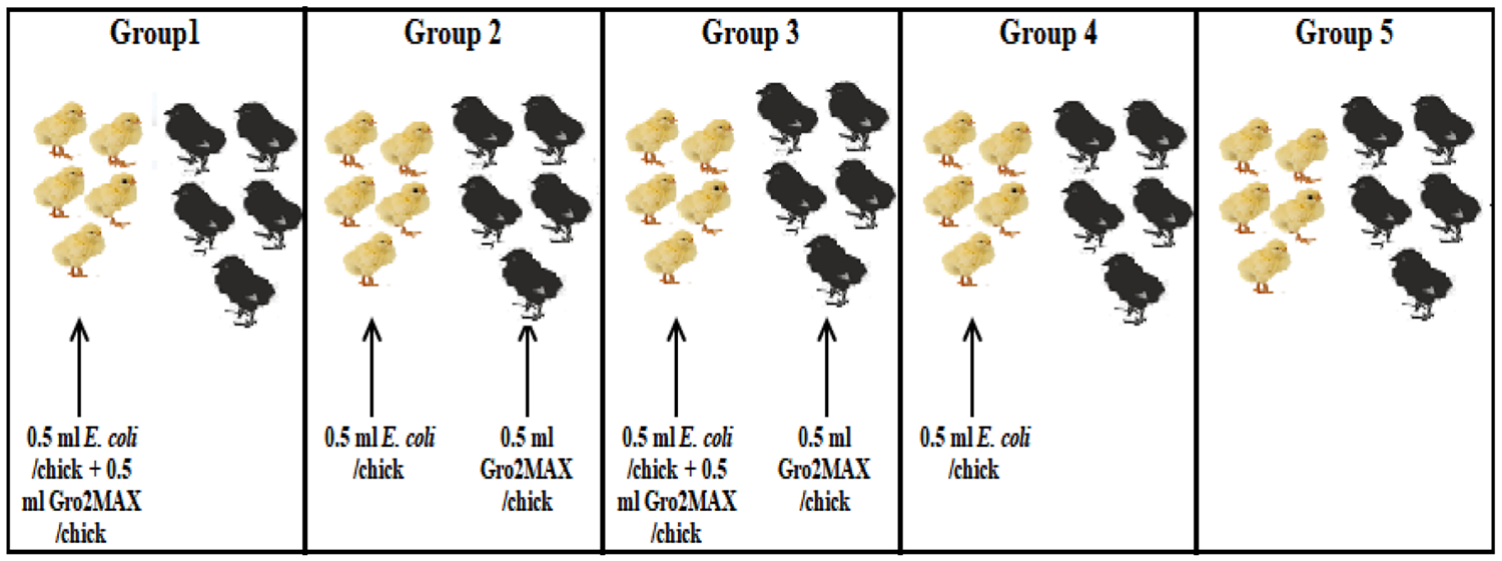
Experimental design of Gro2MAX product supplementation and *E. coli* challenge

Fifty experimental chicks were divided into 5 groups (10 chicks/group), and each bird in each group was individually enumerated. Chicks in each group of 1, 2, 3, and 4 were subdivided into five chicks not challenged (S = susceptible) and five chicks challenged (I = infected) with highly virulent ESBL-producing serotypes from the obtained isolates. Group 5 was not divided and remained a negative control (Fig. 1). Only five chicks in groups 1, 2, 3, and 4 (I = infected) were challenged orally in the crop using a needleless sterile syringe with 0.5 ml containing (10^6^ CFU/ml) according to Ceccarelli et al. [6] on 3rd day of age as the main target of the experiment was the modality to prevent the shedding of ESBL-producing *E. coli* not monitoring any immunological parameters.

The chicks were given CE product on the 1st day of age before being challenged with ESBL-producing *E. coli*. At two time points (1st and 3rd days of age) before the challenge and four-time intervals following the challenge, the excretion of ESBL-producing *E. coli* was assessed in both (S) and (I) chicks in all groups (6th, 9th, 12th, and 15th days of age).

The chicks were observed daily until the experiment ended on the 15th day of age (12 days post-infection). Any observed clinical signs, mortalities, and PM lesions were recorded. The number of CFU of ESBL-producing *E. coli* (O78) was quantified as described by Ceccarelli et al. [6]with modifications; cloacal swabs were collected from all chicks in all groups using sterile dry cotton swabs at 4 time points (6th, 9th, 12th, and 15th days of age). Each swab was weighed before and after sampling to calculate the amount of the collected feces. Each cloacal swab was suspended in 1 ml sterile saline solution (NaCl, 0.85%) and subjected to a tenfold serial dilution (10^1^–10^5^) in sterile saline solution. A total of 10 µl of each dilution was plated on MacConkey plates containing cefotaxime (1 mg/liter agar) and incubated overnight at 37 °C. The number of CFU of ESBL-producing *E. coli* per gram of feces was calculated.

Euthanasia for birds was performed using a gaseous concentration of 45% carbon dioxide to gently render them unconscious [19]. Then, liver and lung samples were collected from each chick through aseptic isolation for gene expression assay and histopathological examination.

Every 3 days all over the experimental period, random cloacal samples were collected from all birds and subjected to PCR and culture diagnostic schemes to check the flora and confirm the free of any pathogenic infection according to the recommendations of Tarrac [20] and Jackwood [21].

### Quantitative Real-time PCR

qRT-PCR was performed with *E. coli* isolates obtained from each group. RNA extraction was carried out using QIAamp RNeasy minikit (Qiagen GmbH, Germany) according to the manufacturer’s instructions in Biotechnology laboratory, Animal Health Research Institute, Zagazig Branch, Egypt. Real-time PCR amplification reaction mixtures were prepared in a final volume of 20 µL containing10 µL of 2 × Hera SYBR Green RT-qPCR master mix (Willow fort, UK), 1 µL of RT enzyme mix (20), 0.5 µL of each primer of 20 pmol concentration, 3 µL of RNase- and DNase-free water, and 5 µL of RNA template. The primer sequences used for the genes involved in toxin, adhesion, and biofilm formation are shown in Table 1.

**Table 1:** Primers sequences, target gene, and cycling conditions for SYBR green rt-PCR

**Target gene**	**Primers sequences**	**Reverse transcription**	**Primary** **Denaturation**	**Amplification (40 cycles)**			**Reference**
				**Secondary denaturation**	**Annealing** **(optics on)**	**Extension**	
*E. coli*	GACCTCGGTTTAGTTCACAGA	50˚C]	94˚C	94˚C	60˚C	72˚C	[24]
* 16S*	CACACGCTGACGCTGACCA	30 min	15 min	15 s	30 s	30 s	
*rRNA*							
* csgA*	CGGAGTGGATGTTAACGACTGG						[25]
	ATGT33TCGCAGACCCAGTCATTG						
* csgD*	CAAGAGGAAAACTCCAGTAATTGCA						[26]
	AAGTCGAAGAGGAAGGCCATAA						
* cnf-1*	AAGATGGAGTTTCCTATGCAGGAG						[4]
	CATTCAGAGTCCTGCCCTCATTATT						
* hly*	AGATTCTTGGGCATGTATCCT						[27]
	GTGGATACGACGATTACTGTG						
* sfa*	GTGGATACGACGATTACTGTG						[4]
	CCGCCAGCATTCCCTGTATTC						

### Histopathological Examination of Internal Organs

Tissue samples (liver and lung) from each group were collected and fixed in buffer formalin solution (10%) then processed with paraffin embedding technique and stained with hematoxylin and eosin [22].

### Statistical Analysis

Data were edited in Microsoft Excel (Microsoft Corporation, Redmond, WA, USA. A Shapiro–Wilk test was conducted to check for normality as described by Razali et al. [23]. Results of the bacterial count were expressed by log_10_. Tukey HSD test was used to compare infected chick versus susceptible chick per day (1, 3, 6, 9, 12, and 15) for both mean quantification (CFU/gm) of ESBL-producing *E. coli* and fold change. Also, the aforementioned statistical test was used to detect the differences between treated groups and the positive control with the level of significance set at *α* = 0.05. Results were represented as means ± SE, and figures were fitted by the GraphPad Prism software 9.0 (Graph Pad, USA). Statistical significance was set at a *p***-**value less than 0.05.

## Results

### *E. coli* Isolation, Serotyping, and Antimicrobial Susceptibility

*E. coli* was isolated from 39 of 100 examined broiler chickens (39%). Ideal microbiological assays were used to categorize them. Ten serotypes were detected, and O78 was the most prevalent serotype (23.07%), followed by O125, O91, O128, and O158 (12.8% each); O44 (7.69%); O124, O2, and O1 (5.1% each); and O55 (2.5%).

All* E. coli *isolates were tested for their susceptibility to ten different antibiotics. Overall, absolute resistance (100%) was observed towards the β-Lactam antibiotics (amoxicillin, ampicillin, cefotaxime, and cephalexin) followed by tetracycline with a percentage of 80%, sulfamethoxazole-trimethoprim (76.6%), streptomycin (53.3%), neomycin (33.3%), and finally, 10% of the tested isolate which were resistant to colistin sulfate. Ten isolates represented multi-drug resistance patterns to two or more antimicrobial groups (25.6%).

### Detection of the β-Lactamase Gene (blaTEM) in *E. coli* Isolates and Sequencing Data

**Fig. 2:**
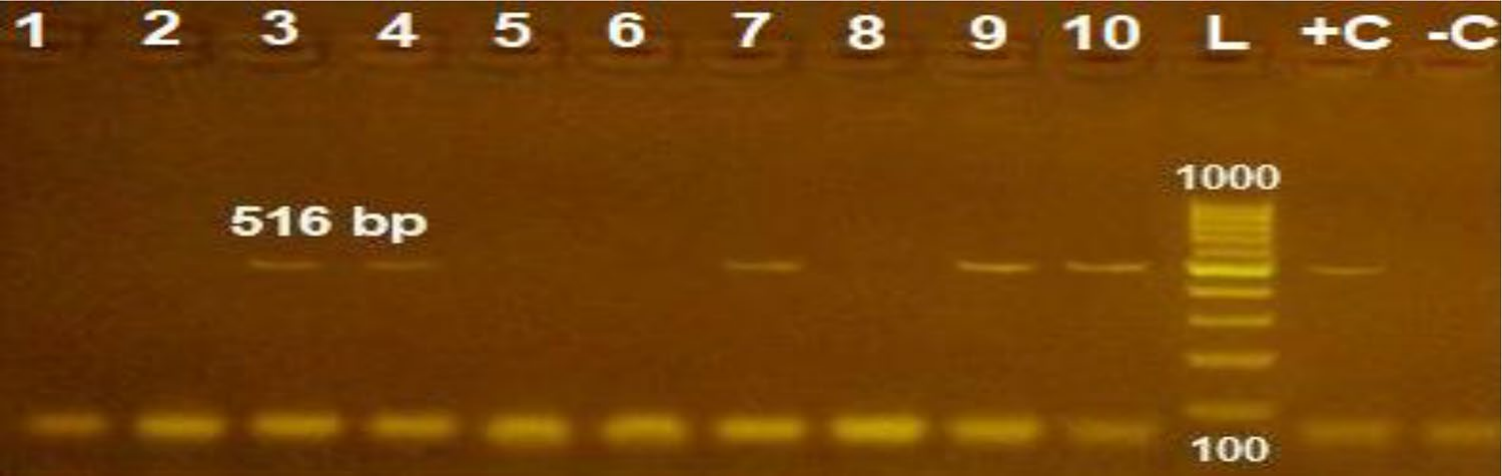
Agarose gel electrophoresis of PCR for amplification products of *bla*_*TEM*_ gene among 10 *E. coli* isolates; lane + C: control positive; lane L: 100-bp ladder (marker); lane -C: control negative

Conventional PCR amplification revealed that 5 out of 10 (50%) of the obtained multidrug-resistant *E. coli *isolates harbored the *blaTEM *gene giving an amplicon size of 516 bp as shown in Fig. 2.

Multidrug-resistant ESBL-producing *E. coli* isolate of serotype O78 selected to challenge and 16S rRNA gene sequencing of its detailed amino acid substitutions in the amplified fragments was deposited to the GenBank under the accession number OQ533606.

### Evaluation of Competitive Exclusion Effect (Gro2MAX Product) in Experimentally Infected Chicks and Its Transcriptional Modulatory Effect

#### Clinical Signs and Postmortem (PM) Lesions of Chicks Under Experiment

Chicks were observed daily until the end of the experiment on the 15th day of age. The observed clinical signs, PM lesions, and mortalities were reported as follows:

The clinical signs appeared by 3rd-day post-infection in group 4 (positive control) only; however, the infected/challenged chicks in groups 1, 2, and 3 showed no clinical signs or mortalities throughout the experimental period. The chicks in the positive control group suffered from dullness, off-food depression, ruffled feathers, and brownish diarrhea. The PM examinations showed Septicemia, severe pericarditis, airsacculitis, and perihepatitis. The 2 ceci were filled with yellowish-to-brownish contents. The mortality rate in the positive control group was started on the 5th day post-infection with a percentage of (30%).

### Competitive Exclusion (Gro2MAX Product) Effect in Reducing ESBL-producing *E. coli* Transmission and Excretion in Experimentally Infected Chicks

**Fig. 3:**
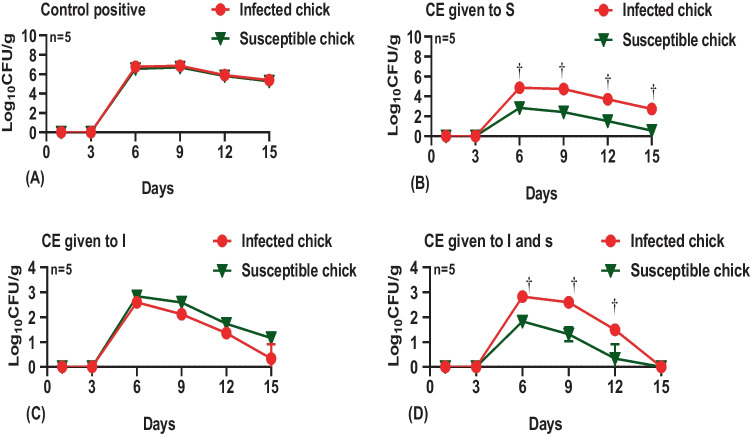
Effect of competitive exclusion (CE) on infected and susceptible birds: control positive (A); CE given to S (B); CE given to I (C); CE given to I and S (D). †*p* < 0.05 infected chick versus susceptible chick. ^a–c^Columns with different superscripts are significantly different

Our findings in Fig. 3 revealed the absence of ESBL-producing *E. coli* at the 2 time points before the challenge. The recorded mean of shedding in the 4 time intervals after the challenge showed a reduction of ESBL-producing *E. coli* in (I) chicks of group 1 and (I & S) chicks of group 3 in comparison with the positive control (group, 4). The mean of shedding of (I & S) chicks in the group (3) which was supplemented with CE product showed a marginally significant reduction all over the time points (6th, 9th, and 12th days of age) though, in the last time point at 15th days of age, the results showed no shedding. The mean shedding detected in (S) chicks (group 1) and (I) chicks (group 2) (both of them non-supplemented with CE) was higher than in chicks treated with CE product. The excretion throughout groups (1, 2, and 3) was decreased at the time points (12th and 15th days) than at time points (6th and 9th days).

Similar effects were observed for the transmission of ESBL-producing *E. coli*, where CE could reduce the ESBL-producing *E. coli* transmission rates. When I chicks were treated alone or in combination with S chicks, CE was effective as the transmission in groups (1&3) was considerably lower when compared with the positive control group.

Overall, non-significant differences were detected between infected and susceptible chick during the whole experimental period in the control positive and the group that CE was given to I (*p* > 0.05). Meanwhile, the group of CE given to S and CE given to I and S showed a significant increase in total bacterial count in the infected chick compared to the susceptible chicks during the whole experimental period (*p* < 0.05) except after 15 days in the group that CE given to I and S showed non-significant differences (*p* > 0.05: Fig. 3A–D).

**Fig. 4:**
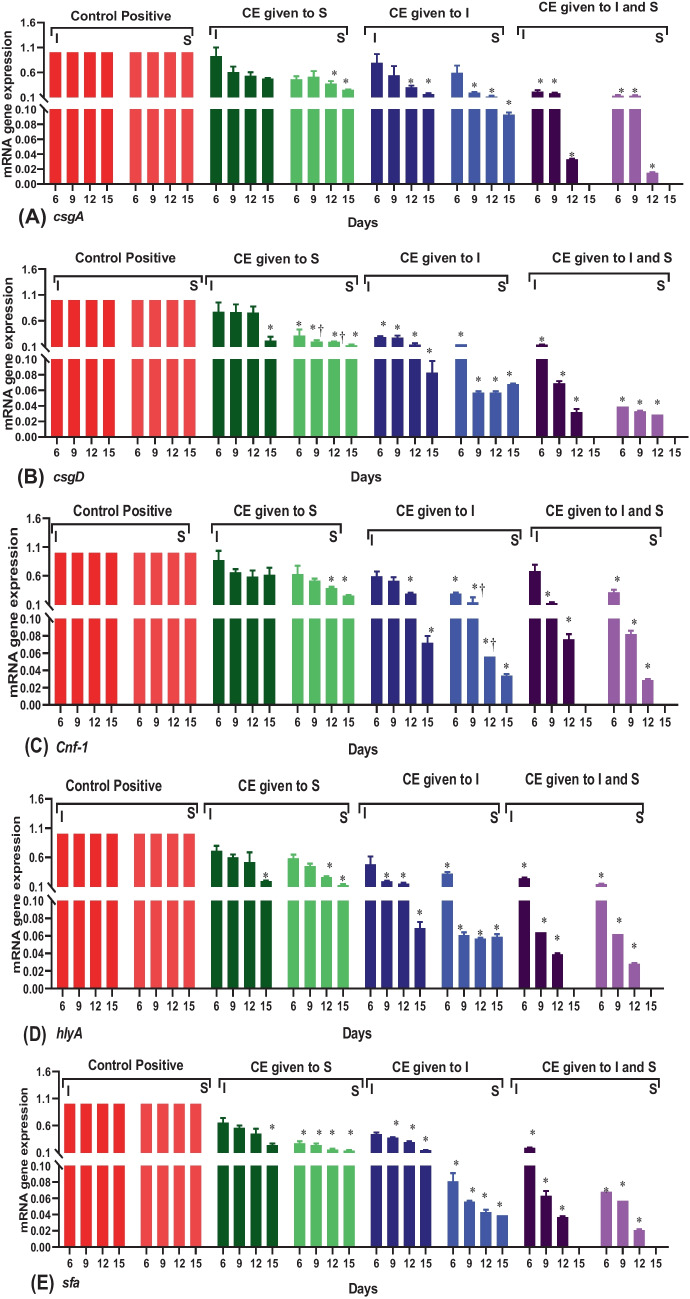
The relative mRNA expression levels of genes related to biofilm production, toxin, and adhesion through obtained *E. coli* isolates in infected and treated groups with different time intervals: **A**: *csgA* gene; **B**: *csgD* gene; **C**: *cnf-1*; **D**: *hly* gene; and **E**: *sfa* gene where control positive represents group 2; CE given to S: group 3; CE given to I: group 4; CE given to S and I: group 5

Regarding molecular evaluation, *csgA* gene expression represented a significant decrease in susceptible groups during all days post-treatment except on the 6th and 9th days. Compared to the infected group, the present results showed a significant decrease (*p* < 0.05) in the aforementioned gene expression in all treated groups except the susceptible group treated by CE (group 3) during all periods considered in addition to the 6th and 9 th days in group 4 (*p* > 0.05; Fig. 4A). Similarly, the lowering trend for *csgD* gene expression was observed in all infected and susceptible treated groups during all study periods (*p* < 0.05) except the period from 6 to 12 days post-treatment in group 3 (*p* > 0.05). Interestingly, there were significant differences between groups 3 and 4 on the 9th and 12th day post-treatment (*p* < 0.05; Fig. 4B)**.** Regarding the expression of *cnf-1 and hlyA *gene expression, the minimum mRNA gene expression was recorded on the 12th and 15th day post-treatment in all studied groups except the expression of the* hlyA* gene was not significant on the 12th day for group 5. Moreover, significant differences were shown between groups 3 and 5 on the 9th and 12th day post-treatment (*p* < 0.05; Fig. 4C and D). For *sfa* gene expression, fold change was significantly lower (*p* < 0.05) in all infected and susceptible treated groups during all periods considered except on the 6th, 9th, and 12th day (*p* > 0.05; Fig. 4E**).**

#### Histopathological Findings

Our microscopical results of experimental chicks in the control negative (group 5) and susceptible chicks (group 1) were similar that exhibited normal hepatic tissue architecture and cellular details in addition to normal pulmonary tissue in all examined chicks. Severe hepatic and pulmonary lesions were seen and demonstrated in both chicks of group 2 (I chicks) and group 4.

**Fig. 5:**
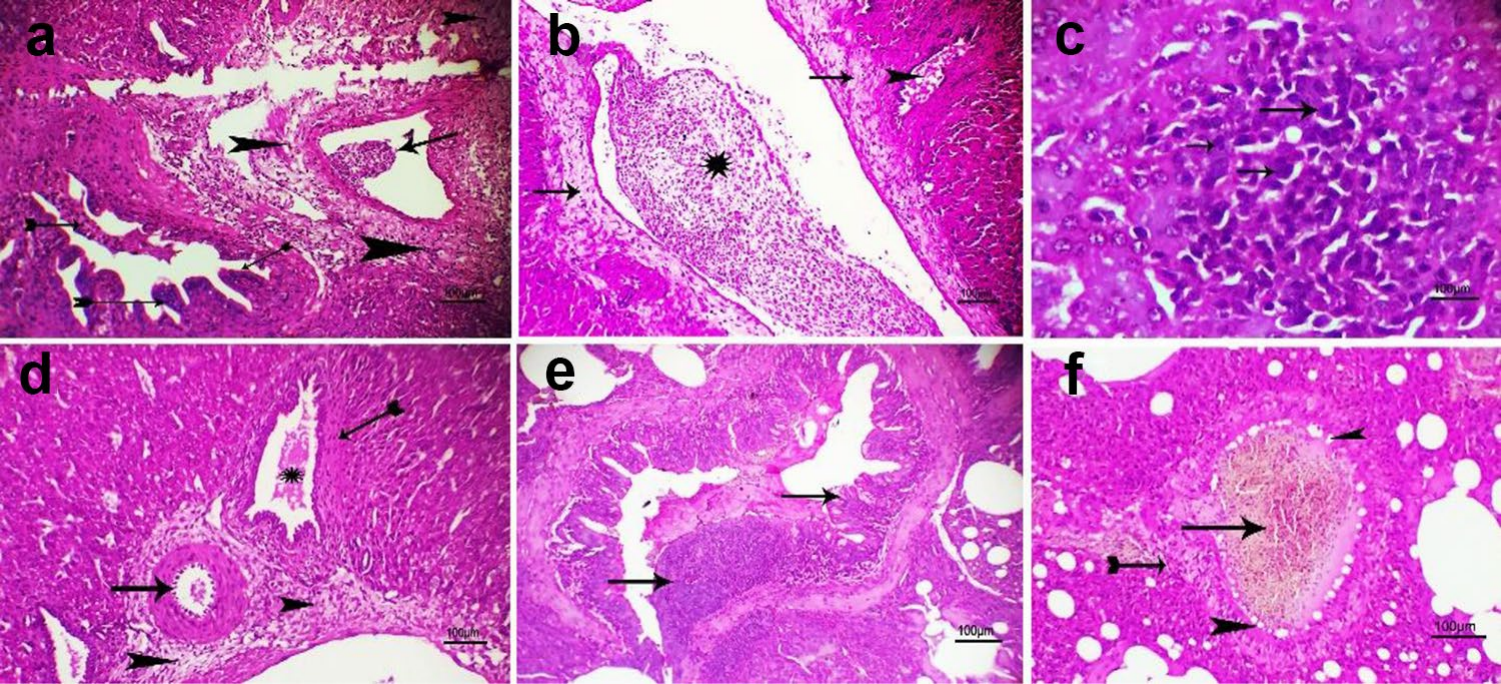
Photomicrograph of H&E stained liver* and lung** of (2I) + and (4) +  + groups at 12th day post-infection with scale bar = 100 µm showing (**a**)* + : hyperplasia of ductal epithelium (tailed arrow) with perivascular fibrosis (arrowhead) and vascular thrombosis (arrow) (**b**)* +  + : severe congestion of hepatic blood vessel (star) with perivascular fibrosis (arrows) and cholestasis (arrowhead) (**c**)* +  + : focal infiltration of hepatic parenchyma with leucocyte cells (arrows) (**d**)* +  + : endotheliosis (arrow) with perivascular fibrosis (arrowhead) in addition to cholestasis (star) and periductal nuclear pyknosis (tailed arrow) (**e**)** + : hyperplasia of bronchial epithelium (arrows) (**f**)** +  + : congestion of blood vessel (arrow) with vacuolation of tunica intema (arrowhead) and perivascular fibrosis

There was thrombus development occasionally, as well as ductal epithelium hyperplasia with or without perivascular fibrosis in the liver (Fig. 5a)**.** Both groups predominantly displayed moderate to severe hepatic vascular congestion, with or without cholestasis and fibrosis (Fig. 5b). Moderate to severe focal infiltration of hepatic parenchyma with leucocyte cells was the most characteristic lesion that appeared in all infected groups (Fig. 5c). In group 4, nuclear pyknosis with chromatin condensation (Fig. 5d) represents endotheliosis with periductal coagulative necrosis. However, both groups had congested lungs, and only one chick in group 2 had hyperplasia of the bronchial epithelium (Fig. 5e). Vacuolation of tunica intema with/without fibrosis (Fig. 5f**)** was noticed in both groups.

**Fig. 6:**
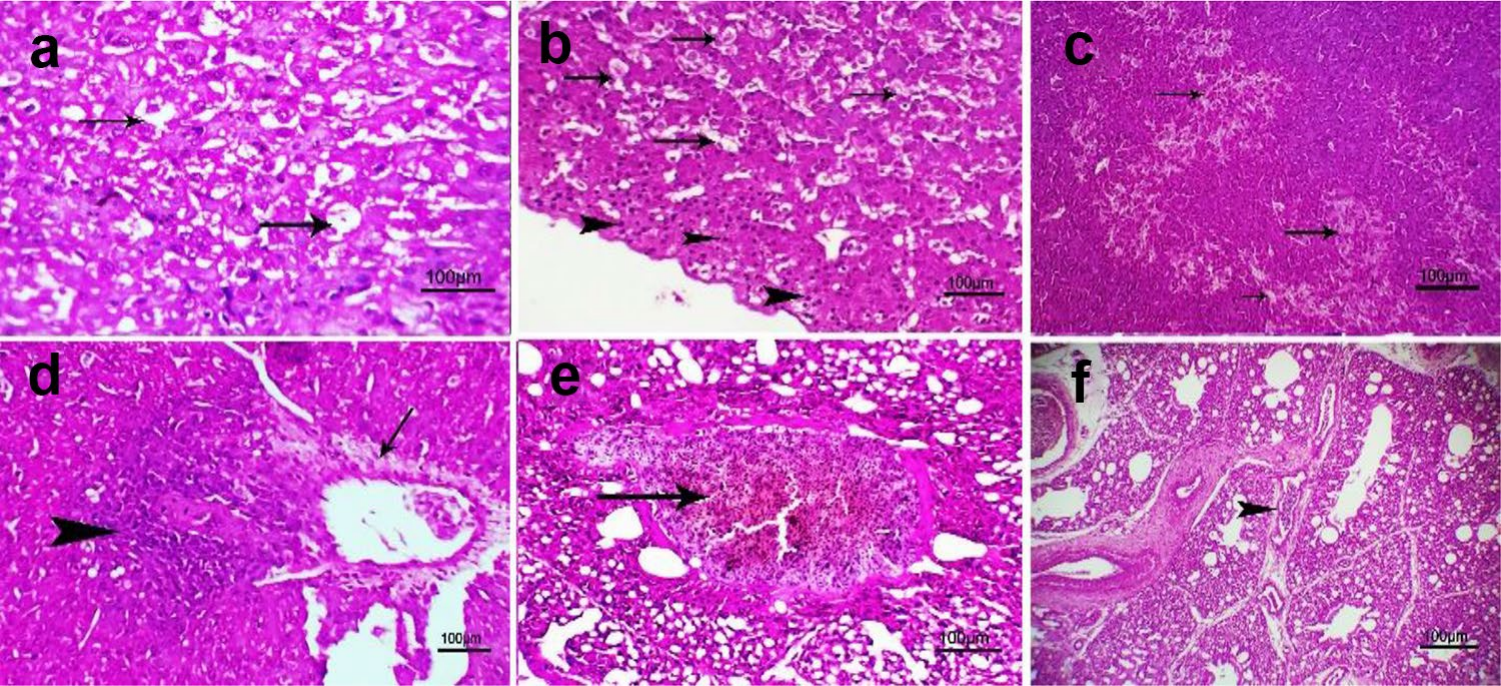
Photomicrograph of H&E stained liver* and lung** of (1I) + and (3I) +  + groups at 12th day post-infection with scale bar = 100 µm showing (**a**)* + : focal hydropic degeneration of some hepatocytes (arrows) (**b**)* + : congestion of hepatic sinusoids (arrows) with subcapsular coagulative necrosis (pyknosis) (arrowhead) (**c**)* +  + : focal areas of caseous necrosis (arrows)(d)* +  + : perivascular fibrosis (arrow) with leucocyte cells infiltration (arrowhead) (**e**)** + : congestion of blood vessel (arrow) (**f**)** +  + : mild congestion of blood vessel (arrow)

Previously mentioned lesions in infected and non-supplemented groups with CE product were diminished and became mild in some chicks of groups 1 and 3 (I chicks) and even disappeared. The liver showed focal hydropic degeneration of some hepatocytes (Fig. 6a**),** congestion of the hepatic sinusoids with subcapsular coagulative necrosis (pyknosis) (Fig. 6b**),** focal areas of caseous necrosis (Fig. 6c), perivascular fibrosis with leucocyte cells infiltration (Fig. 6d**),** congestion of blood vessels (Fig. 6e), and mild congestion of blood vessel (Fig. 6f).

**Fig. 7:**
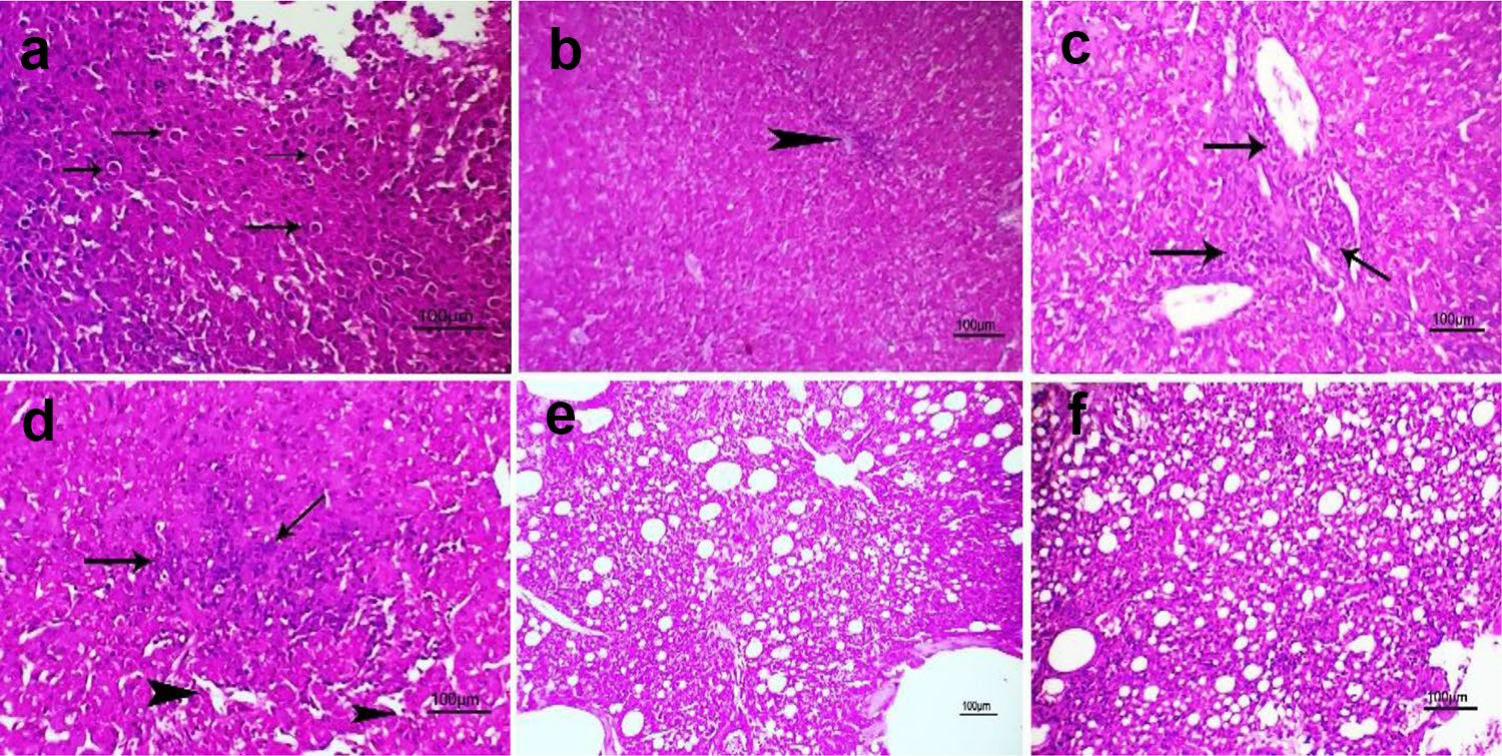
Photomicrograph of H&E stained liver* and lung** of (2S) + and (3S) +  + groups at 12th day post-infection with scale bar = 100 µm showing (**a**)* + : individualization of some hepatocytes ( arrows) (**b**)* +  + : mild focal cellular infiltration (arrow) (**c**)* +  + : perivascular cellular infiltration (arrows) (**d**)* +  + : focal cellular infiltration (arrows) with dilated sinusoids (arrowhead) (**e**)** + : apparently normal pulmonary tissue (**f**)** +  + : minimal cellular infiltration with normal pulmonary tissue

The livers of groups 2 and 3 (S chicks) displayed individualization of certain hepatocytes (Fig. 7a)**,** mild localized cellular infiltration (Fig. 7b)**,** perivascular cellular infiltration (Fig. 7c), and focal cellular infiltration with dilated sinusoids (Fig. 7d)**.** The lung exhibited normal pulmonary tissue (Fig. 7e**)** and minimal cellular infiltration with normal pulmonary tissue (Fig. 7f).

## Discussion

Avian colibacillosis is one of the serious diseases in chickens caused by *Escherichia coli*, which is considered one of the principal causes of morbidity and mortality, associated with heavy economic losses to the poultry industry documented by several earlier investigations [28, 29]. It’s essential to constantly monitor the rising rates of antibiotic resistance among *E. coli* because they are a significant healthcare issue [30–32]. The widespread occurrence of ESBL *E. coli* (extended-spectrum-lactamases *Escherichia coli*) in broiler production constitutes a growing issue for veterinary medicine and public health [33, 34]. ESBL *E. coli* has the potential to enzymatically deactivate beta-lactam antibiotics [35], which imparts resistance to several antibiotics, including 3rd and 4th generation cephalosporins, which are categorized as “critically important antimicrobials” [36]. Broilers can harbor ESBLs and release them during the food manufacturing process [37, 38].

For this reason, we established the current study to shed light on the antibiotic resistance profiles of *E. coli* isolated from broiler chickens in Egypt and evaluation the alternative applied assay to overcome this resistance.

Herein, the overall incidence level of *E. coli* (39%) from broilers was nearly similar to those detected by Sharada et al. [39] in India, Literak et al. [40] in the Czech Republic, and Amer et al. [41] in Egypt. Higher incidence rates, with percentages of 80 and 63.6%, respectively, were previously reported by Eid and Erfan [42] in Egypt and Al-Arfaj et al. [43] in Saudi Arabia. On the other hand, a lower incidence of 15.8% was detected by Momtaz et al. [44]. The variation in the isolation rates in this study in comparison with the previous studies is owing to the impact of several conditions such as sampling techniques, isolation procedures, seasonal variation, and geographic area.

From the obtained results, it was revealed that O78 was the most prevalent serotype as previously stimulated by Enany et al. [45] in Egypt, Halfaoui et al. [46] in Algeria, and Ibrahim et al. [47] in Jordan.

Antimicrobial resistance has developed widely as a result of the indiscriminate use of antimicrobial agents, which has gotten a lot of national and international attention [30]. The current resistance phenotypes with multidrug resistance (MDR) recorded in this study are similar to the data reported from another regional study [48] in Egypt as well as worldwide studies such as Halfaoui et al. [46] in Algeria, Ibrahim et al. [47] in Jordan, Singh et al. [30] in India**.**

Penicillins, sulfonamides, tetracyclines, and aminoglycosides have a high resistance rate, which may be due to the fact that these drugs are the oldest used to treat infectious diseases [49]. It is expected that a high level of resistance would have emerged over time. The frequencies and patterns of antimicrobial resistance may vary depending on time, region, serovar, the particular farm, type of chicken (layer versus broiler), and antimicrobial agent.

Of interest, ten *E. coli* isolates (25.64%) were resistant to at least three of the ten tested antimicrobial agents, making them MDR. This finding was in agreement with that reported in Poland [50] and in Tanzania [51] representing a significant disease burden in Egypt.

Of relevance is the high incidence (50%) of ESBL producers detected among the collection of MDR *E. coli* isolates. A similar incidence rate in chicken samples has been recognized previously in Egypt, especially in northern cities [52]. This described extraordinary prevalence of ESBL producers in chicken signifies a great influence on the high detection level of these superbugs in human samples. As chicken-offal (liver, gizzard, bone, kidney, feet, and heart) is a widespread fast food in Egypt and numerous developing countries since it is cheap, effortlessly cooked, and a potential supplier of proteins [53].

The use of competitive exclusion (Gro2MAX; a commercial probiotic product) in this study decreased the excretion and transmission of ESBL-producing *E. coli* in vivo. Clinical symptoms, PM lesions, and death rate were similar to those observed by Alexander et al. [54] and Abd Elatiff et al. [55]**.** The acute cases of* E. coli* in experimental infection showed the possible isolation from 6 h to 3 days after infection as previously recorded by by Eid et al. [56]

The continued supplementation of CE products within a short period after hatching may be a helpful solution to minimize ESBL-producing bacteria in broiler farms [8]. Our results revealed that the mean of ESBL-producing *E. coli* shedding was minimized in (I chicks; group 1) and (I and S chicks; group 3) when compared to a positive control (group 4). Even though the mean shedding observed in (S) chicks (group 1) and (I) chicks (group 2), neither of which received CE supplements, was reduced, it remained higher than in chicks receiving CE product treatment.

This may be due to the CE depending more on the reduced shedding of infected birds than on the susceptibility reduction of susceptible birds [6]**. **According to our research, there was a noticeable decrease in the excretion of broiler intestines by *E. coli*-producing ESBL, and this finding agreed with that recorded by la Ragione et al. [57], Nuotio et al. [58], and Daehre et al. [59]. Additionally, a slight reduction in the transmission of *E. coli*-producing ESBLs was detected when compared to the positive control group, which was similar to the study conducted by Dame-Korevaar et al. [8].

The ability to produce biofilms gives bacteria extraordinary survival advantages, enabling defense against mechanical, chemical, and biological threats. Additionally, it promotes the spread of genes encoding for antimicrobial resistance among bacteria [60]. The protected bacteria by a biofilm in a water pipeline or on animal surfaces are less vulnerable to detergent and antibiotic treatments.

In detail, biofilm formation restricted the diffusion of beta-lactam antibiotics through the matrix, (i) communication between the beta-lactams and the biofilm matrix (polymer and cells), (ii) levels of metabolic activity within the biofilm, and (iii) enzyme-mediated resistance leading to the synthesis of beta-lactamase [61].

The majority of *E. coli* isolates from three separate chains of an integrated poultry enterprise in Italy were able to produce biofilm, demonstrating the severity of this problem with *E. coli*-producing ESBLs [62, 63].

Herein, we demonstrated the relationships between the development of biofilms and several virulence factors that encode adhesins and toxin synthesis that might be involved in the initial bacterial adhesion process to surfaces and the progress of the mature biofilm which resulted in strong expression of beta-lactam resistance.

Biofilm formation was associated with the presence of *the CsgBAC* operon and encoded by the csgA-D genes. Additionally, the presence of *hly*, *cnf-*1, and *sfa* genes enhances biofilm production across elevation of toxin generation and fimbrial expression, which facilitates adhesion and permits biofilm synthesis [63].

Investigating the expression of these identified genes allowed us to improve the knowledge regarding the genetic factors influencing the biofilm formation ability of *E. coli*-producing ESBLs. Thus we scrutinized the transcriptional effect of competitive exclusion (Gro2MAX product) on biofilms of *E. coli* isolates at different experimental periods. We have displayed that the utmost downregulation in biofilm-associated *csgA *and* csgD* genes was recorded in *E. coli* isolates, and there was a significantly higher efficiency in the downregulation of *cnf-1, hly*, and *sfa* genes over the control, especially at 15th day post-infection. This finding is strongly correlated to prior studies that detected the ability of probiotics to suppress* E. coli* biofilm formation by regulating the expression of involved genes [64, 65].

Histopathological findings showed severe hepatic and pulmonary lesions in group 2 (I chicks) and group 4 (positive control) on the basis of similarity between chicks of both groups in treatments and time of exposure in addition to similarity in breed, species, and age of chickens with circumstances of rearing. Each group represented a control positive for other treated shared groups.

These lesions were diminished and became mild and even disappeared in some infected chicks with *E. coli* (O78) and supplemented with CE (group 1; I chicks and group 3; I chicks). Mild minimal lesions with reasonably normal lung tissue were discovered in chicks treated with CE (group 3) but not infected ones (group 2). Probiotics (CE products), according to the study’s conclusive findings, can bolster and repair the tissues of the internal organs.

Our findings were consistent with other research reported by Abd Elatiff et al. [55] who noted an improvement in the histopathological sections following the application of probiotics.

## Conclusion

Using the experimental model for exposure, a commercial CE culture (Gro2MAX product) considerably diminishes the cecal colonization of extended-spectrum β-lactamase *Escherichia coli* isolates in broilers with significant minimization and downregulation for involved genes in biofilm synthesis. Therefore, the application of the Gro2MAX product was of great value in protection against broadcast and excretion of ESBL-producing *E. coli* in broiler chickens.

## Data Availability

All data used have been included in the manuscript.
